# Remote Plasma-Induced Synthesis of Self-Assembled MoS_2_/Carbon Nanowall Nanocomposites and Their Application as High-Performance Active Materials for Supercapacitors

**DOI:** 10.3390/nano12081338

**Published:** 2022-04-13

**Authors:** Jin-Ha Shin, Yong-Sup Choi, Hyun-Jae Park

**Affiliations:** Institute of Plasma Technology, 814-2 Osickdo-dong, Gunsan 573-540, Jeollabuk-do, Korea; margth666@kfe.re.kr (J.-H.S.); yschoi@kfe.re.kr (Y.-S.C.)

**Keywords:** carbon nanowall, remote plasma, hydrogen radical, MoS_2_, nanocomposite, supercapacitor

## Abstract

The objective of this study is to investigate the synthesis and influence of MoS_2_ on carbon nanowalls (CNWs) as supercapacitor electrodes. The synthesis of MoS_2_ on CNW was achieved by the introduction of hydrogen remote plasma from ammonium tetrathiomolybdate (ATTM) without deterioration of the CNWs. The topographical surface structures and electrochemical characteristics of the MoS_2_–CNW composite electrodes were explored using two ATTM-dispersed organic solvents—acetonitrile and dimethylformamide (DMF). In this study, CNW and MoS_2_ were synthesized using an electron cyclotron resonance plasma. However, hydrogen radicals, which transform ATTM into MoS_2_, were provided in the form of a remote plasma source. The electrochemical performances of MoS_2_–CNW hybrid electrodes with various morphologies—depending on the solvent and ATTM concentration—were evaluated using a three-electrode system. The results revealed that the morphology of the synthesized MoS_2_ was influenced by the organic solvent used and affected both the electrochemical performance and topographical characteristics. Notably, considerable enhancement of the specific capacitance was observed for the MoS_2_ with open top edges synthesized from DMF. These encouraging results may motivate additional research on hybrid supercapacitor electrodes and the rapid synthesis of MoS_2_ and other transition metal dichalcogenides.

## 1. Introduction

In accordance with environmental regulations and energy policies, a contemporary technological and industrial society demands the use of environmentally friendly energy sources and storage systems on a major scale to reduce fossil fuels. Currently, the most widely used energy storage devices are nickel-metal hydride and lithium secondary batteries. However, in secondary batteries, the voltage drop and service life are shortened during high-power discharge; therefore, they must be replaced every two–three years. As a result, supercapacitors have recently attracted considerable attention as a possible solution to this limitation.

Indeed, supercapacitors possess high power density, rapid charge/discharge properties, and a very long charge–discharge life of more than 500,000 cycles. Furthermore, supercapacitors play an important role of filling the gap between batteries and conventional capacitors by providing higher power density than that of batteries and higher energy density than that of conventional capacitors (e.g., electrolyte capacitors or metalized film capacitors) [1,2].

As is well known, supercapacitors can store electrical energy through two fundamentally different mechanisms: pseudocapacitors use potentially available chemical energy, based on electron transfer by faradaic redox reactions between electrochemically active materials with several oxidation states of metal atoms present in the electrode and electrolytes [3,4]. Although a large amount of charge can be stored by faradaic electrochemical processes, pseudocapacitors are limited by potential delay during charging/discharging due to the passage of charge across the double layer and lack of cycling stability [5,6]. On the other hand, electrical double layer capacitors (EDLCs) work by the electrostatic adsorption of ions in the electrolyte on an active material surface to form a Helmholtz interface [7,8]. Thus, the active materials of EDLCs require a large surface area and have the advantages of response speed and chemical stability during cycling [9].

Carbon allotrope materials have been widely studied as candidates for EDLC electrodes due to their unique characteristics including chemical stability, excellent electrical conductivity, cost efficiency, and remarkable specific area. Therefore, diverse carbon-based materials—including activated carbon [10,11,12], carbon nanotubes [13,14], carbon nanowalls (CNWs) [15,16], and graphene [14,17,18]—have been developed as EDLC electrode materials. Although EDLCs possess better properties in terms of their life cycle, rapid charging–discharging, and stability, they generally display lower capacitance compared to pseudocapacitors, due to the difference in their charge storage mechanisms. Therefore, numerous efforts have been made worldwide to overcome this issue.

**Scheme 1:**
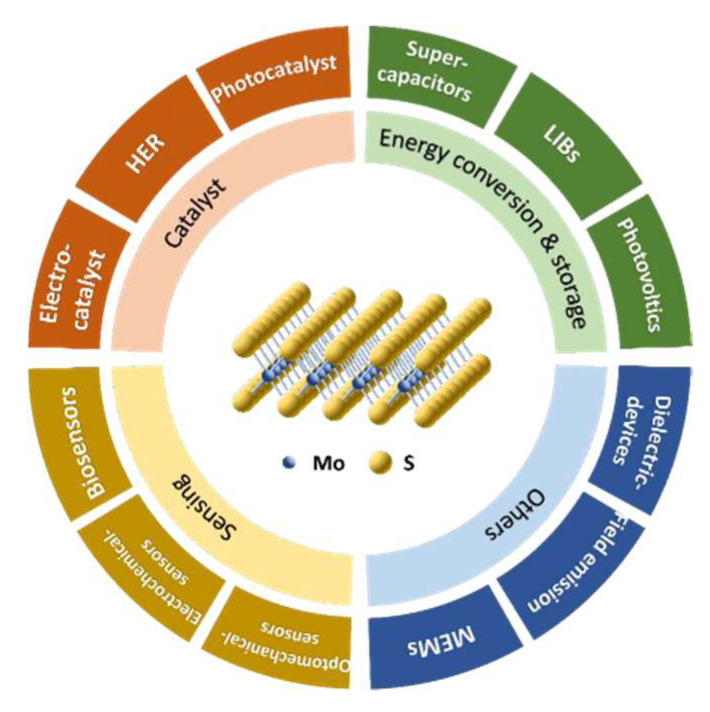
Illustration of MoS_2_ applications in various fields.

Two-dimensional transition metal dichalcogenides (TMDs) such as MoS_2_, WS_2_, MoSe_2_, TiS_2_, NbS_2_, and VS_2_ have been recently suggested as promising candidates for supercapacitor electrode materials, due to their unique electrochemical properties for charge storage attained by providing metal atoms in diverse oxidation states [19,20,21,22,23,24,25]. Among them, MoS_2_ is the most widely researched TMD material, and it has been established as a promising material for variety of applications, including catalysis [26,27], sensing [28,29], and energy conversion and storage [4,15,19,22,23,30,31,32,33,34,35] (Scheme 1). This is due to the fact that the oxidation states of the Mo-centered atom in the S–Mo–S layered structure can be tuned in the range of +2–+6 to provide the required pseudocapacitive characteristics. Various synthesis methods of MoS_2_ have been developed—such as mechanical exfoliation, exfoliation in the liquid phase, and sputtering as top-down approaches and physical vapor deposition, solution chemical processes, chemical vapor deposition, and atomic layer deposition as bottom-up approaches [36,37]. MoS_2_ can also be prepared by thermal decomposition of ammonium tetrathiomolybdate ([NH_4_]_2_MoS_4_; ATTM). 

The thermal dissociation reaction of ammonium tetrathiomolybdate is presented below [24]:

[NH_4_]_2_[MoS_4_] ↔ MoS_3_ + 2NH_3_ + H_2_S (thermal decomposition at 155–280 °C).

MoS_3_ ↔ MoS_2_ + S (decomposed again at 300–820 °C)

MoS_3_ + H_2_ ↔ MoS_2_ + S (under hydrogen atmosphere, ~450 °C)

With these facts in mind, in this study, a MoS_2_–CNW hybrid-structured electrode (e.g., the hetero-structure with combined MoS_2_ and CNW) for supercapacitors was designed and developed. The combination of MoS_2_ and CNW endow this electrode with four main advantages over its conventional counterparts: (1) rapid charge transport between MoS_2_ and CNW by intimate contact between the 2D materials; (2) considerable reduction of material usage and improved energy density owing to the contribution of faradaic and non-faradaic capacitances; (3) enhancement of the charge–discharge rates by shortened electron and ion transport lengths and improved electrical conductivity of the layered MoS_2_ and CNW structure; and (4) improvement of the electrochemical performance from the highly exposed surface. 

Incorporation of carbon-based conductive additives in MoS_2_ encompassing above advantages is expected to significantly improve both cycle stability and rate capability of supercapacitor electrode. 

Although MoS_2_–carbon allotrope hybrid-structured electrodes have been extensively researched, the long synthesis time (typically 24 or longer) and complex steps limit their scalability. In this study, the MoS_2_ on CNW structure was synthesized with hydrogen radicals (using a remote plasma) to introduce a MoS_2_–CNW hybrid electrode into a supercapacitor without compromising the electrochemical properties of the CNW. To the best of our knowledge, this is the simplest and most effective method reported to date. In this method, the hydrogen radicals were anticipated to promote the ATTM–MoS_2_ conversion process by unpaired valence electrons, which make the radicals highly chemically reactive [38]. The remote plasma process can supply reactive hydrogen radicals to the ATTM and is a possible method to transform ATTM into MoS_2_ in the absence of a heating system (without damaging the CNW scaffold by ion impact) in a very short synthesis time. To synthesize the CNW scaffolds and MoS_2_, an electron cyclotron resonance (ECR) plasma system was employed. ECR plasma is the most suitable source for the rapid synthesis of CNW, since it has the highest electron temperature and electron density among the existing plasma sources. 

The encouraging results obtained in this study may motivate further research on CNWs and their hybrid structures for targeting high-power EDLC applications. The crystallinity of the synthesized MoS_2_ was confirmed using Raman spectroscopy. The fabricated CNW–MoS_2_ hybrid electrodes were then evaluated as EDLC electrodes using cyclic voltammetry (CV) and electrochemical impedance spectroscopy (EIS) analyses.

## 2. Materials and Methods

MoS_2_-combined CNW electrodes for supercapacitor applications were fabricated to improve the electrochemical capacitive properties using hydrogen-introduced remote ECR plasma in the absence of a heating system. Prior to the synthesis process, a Ni transition metal substrate (99.95%, Alfa Aesar) was prepared in a 5% sulfuric acid aqueous solution to remove the native oxide film on the substrate surface. The substrate was then rinsed with deionized (DI) water. Organic contaminants were removed by repeated 30 min sonication cycles in acetone, followed by rinsing with DI water. 

During the CNW synthesis process, hydrogen was supplied as a carrier gas to decompose the methane reactive precursor, eliminate any residual organic contamination and native oxide films, and create active sites for nucleation seeds. The ECR plasma was generated using 1 kW microwaves with a fixed hydrogen flow rate of 100 sccm and a methane flow rate of 50 sccm for a growth time of 30 min. 

ATTM was dispersed in two different polar organic solvents—Dimethylformamide (DMF; [CH_3_]_2_NC[O]H) and acetonitrile, as dispersion and dissolution media of ATTM—at weight percentages of 0.1, 0.5, 1, and 2 wt.%. The mass loading of ATTM, MoS_2_ source, on CNW scaffold was approximately 0.02–0.4 mg. The samples were then labeled according to the type of solvent in which ATTM was dispersed and the ATTM concentration in weight percent (e.g., 0.1AM–CNW for the 0.1 wt.% ATTM-dispersed acetonitrile MoS_2_–CNW sample and 2DM–CNW for the 2 wt.% ATTM-dispersed DMF MoS_2_–CNW sample). The ATTM solution was then sonicated with a bar-type sonicator for over one hour. The solution was drop-casted onto the prepared CNW substrate and dried for 10 min in a 100 °C vacuum oven. The prepared substrates were treated with remote plasma with a fixed hydrogen flow rate of 100 sccm and 1 kW microwave power for 30 min.

The morphological properties were characterized using field emission scanning electron microscopy (FE-SEM) and Raman spectroscopy with an excitation wavelength of 532 nm. X-ray photoelectron spectroscopy (XPS) analysis was conducted using an Al Kα X-ray source. An elemental analyzer was employed to evaluate the deposition rate of the CNWs on the Ni substrates. Electrochemical measurements were conducted in a 6 M KOH electrolyte diluted in DI water. For the three-electrode system, Pt wire and Ag/AgCl were used as the counter and reference electrodes, respectively. Finally, CV measurements were conducted in the potential range of −1–0.2 V at various scan rates in the range 10–1000 mV s^−1^, while EIS was conducted in the frequency range of 100 mHz–100 kHz.

## 3. Results and Discussion

### 3.1. Scanning Electron Microscopy (SEM) Analysis

**Figure 1:**
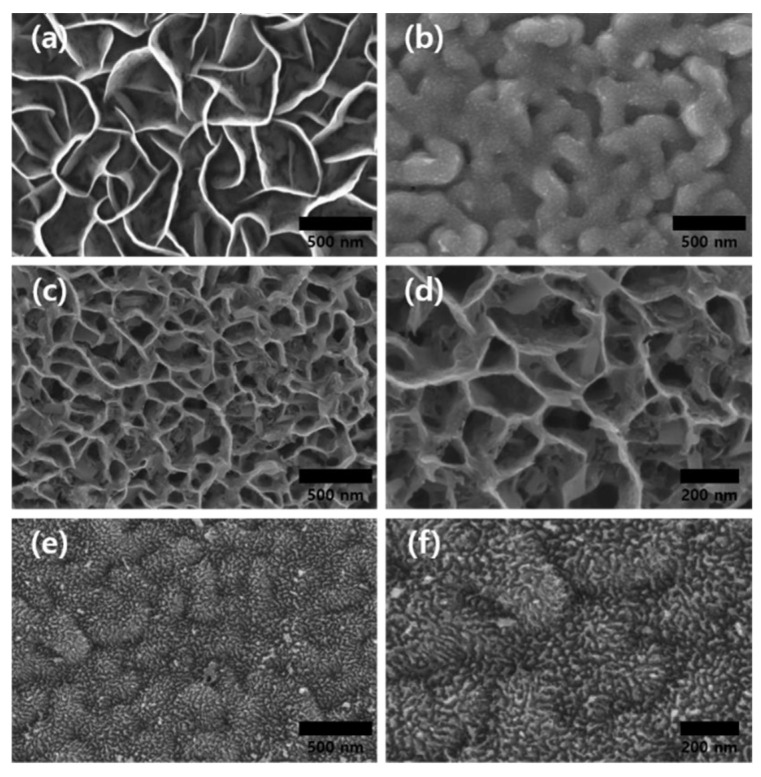
Top view images of the (**a**) pristine carbon nanowall (CNW), (**b**) 500 °C heat-treated ammonium tetrathiomolybdate (ATTM) under hydrogen atmosphere, (**c**) dimethylformamide (DM)–CNW (×50,000), (**d**) DM–CNW (×100,000), (**e**) acetonitrile (AM)–CNW (×50,000), (**f**) and AM–CNW 09 (×100,000).

SEM analysis revealed well-defined nanoflake morphologies of the hydrogen-remote plasma-treated ATTM synthesized over CNW substrate with DMF and acetonitrile organic solvents, compared to those of the pristine CNW and 500 °C heat-treated samples under hydrogen atmosphere (Figure 1). Moreover, as depicted in Figure 1c–f, the MoS_2_ flakes showed different morphologies depending on the organic solvent used. Thus, for the DM–CNW sample, MoS_2_ formed an interconnected nanowall network structure, whereby the CNW was most likely provided as structural scaffolds. This resulted in a plurality of pores of approximately 100 nm forming between each carbon wall. On the other hand, a fine MoS_2_ nanoflake structure with dozens of nanometers was observed for the AM–CNW sample. Both results were predicted to enhance the electrical double-layer capacitance by increasing the surface area and wettability of the electrodes. The electrolyte–electrode contact area is significantly dominated by the capillary force derived from the wetting of the porous surface, which depends on the relative magnitudes of the capillary and resistance forces according to the Wenzel and Cassie–Baxter models [39,40,41]. Therefore, the pore size distribution is the most important factor for increasing the effective surface area. Compared to that of the pristine CNW, the 200 nm average distance between each nanowall was distinctively decreased to 65 nm for the DM–CNW samples. The SEM results revealed that the electrolyte contacted the interior carbon walls and confined air simultaneously, forming the Cassie–Baxter state for the pristine CNW sample. On the other hand, the effective contact area between the electrolyte and MoS_2_–CNW electrode was considerably enhanced, forming the Wenzel state.

### 3.2. Raman Spectroscopy Analysis

Raman spectra were examined according to the ATTM concentration and solvent type to evaluate the crystallinity and lattice structure of the hybrid electrodes, using a laser with a wavelength of 532 nm. 

**Figure 2:**
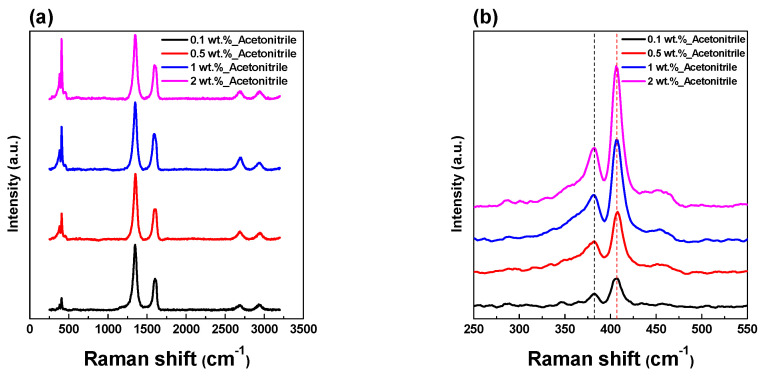

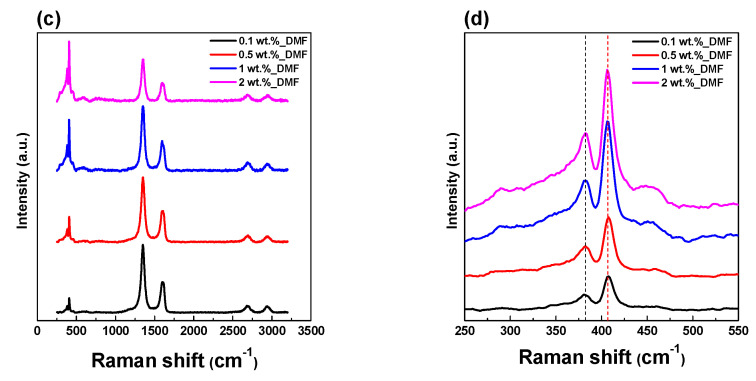
Raman shifts of the hydrogen radical-treated MoS_2_–CNW samples depending on the or-ganic solvent type and ATTM concentration: (**a**) MoS_2_–CNW synthesis using acetonitrile, depending on the ATTM concentration; (**b**) enlarged spectra of the $E_{2g}^{1}$ and A_2g_ modes in (**a**); (**c**) MoS_2_–CNW syn-thesis using dimethylformamide (DMF), depending on the ATTM concentration; and (**d**) enlarged spectra of the $E_{2g}^{1}$ and *A_2g_* modes in (**c**).

In the normalized Raman spectra (Figure 2a,c), peaks corresponding to MoS_2_ and CNW appeared for both the AM–CNW and DM–CNW samples. The characteristic D (1343 cm^−1^), G (1595 cm^−1^), and 2D (2685 cm^−1^) bands indicate that carbon-based materials were obtained for all of the samples [42,43,44]. Moreover, the disorder-induced D band indicates the presence of defects—such as substitutional heteroatoms, vacancy-like defects, and boundary defects—in the crystals. The Raman active G band is associated with the in-plane vibrational mode of the sp^2^ carbon atom constraints, while the 2D band is associated with the two-phonon double-resonance process, which is a function of the graphene layers [34].

The two distinctive Raman lattice vibration modes of MoS_2_ at 382 and 407 cm^−1^ were assigned to the $E_{2g}^{1}$ and *A_1g_* modes, respectively, indicating the formation of 2H–MoS_2_ from the hydrogen radical process (Figure 2c,d) [45,46,47]. Figure 2b,d show the intensity variation of the $E_{2g}^{1}$ and *A_1g_* modes according to the ATTM concentration in acetonitrile and DMF. For both modes, the peak intensity increased with increasing ATTM concentration, demonstrating that the quantity of MoS_2_ in the hybrid electrodes also increased. Moreover, after 30 min of exposure to hydrogen radicals, the vibration modes of CNW and MoS_2_ were still present, indicating that structural deformation or degradation of MoS_2_ and CNW did not occur [48]. These results confirmed that MoS_2_ can be produced efficiently (even at high ATTM concentrations) using a hydrogen-radical-induced synthesis technique.

For the hydrogen-radical-synthesized MoS_2_ samples in acetonitrile and DMF, characteristic Raman lattice vibration modes at 382, 407, and 454 cm^−1^ were observed, which were assigned to the $E_{2g}^{1}$, *A_1g_*, and longitudinal acoustic phonon modes of 2H–MoS_2_, respectively (Figure 2b,d) [49,50,51].

Due to the increased surface area, opening of the additional van der Waals gaps, and active dangling bonds provided to the electrolyte, the surface shape and orientation of the supercapacitor electrodes have significant implication on the electrochemical performance. Accordingly, the surface information of the synthesized MoS_2_ samples can be provided by the relative intensity ratio of the $E_{2g}^{1}$ and *A_1g_*, bands [45,52]. For the AM–CNW and DM–CNW samples, the relative Raman intensities of $E_{2g}^{1}$ and *A_1g_* were 0.64 and 0.45, respectively, suggesting that edge-orientated MoS_2_ was obtained from the DM–CNW but not the AM–CNW samples. Edge-oriented MoS_2_ has the advantage that reactive transition metal atoms are exposed along the sheet edges. These atoms contribute toward increasing the capacitance of the hybrid electrodes by intercalation of ions in the electrolyte and reversible redox reactions between the 4+–3+ valence states of Mo [45].

### 3.3. XPS Analysis

**Figure 3:**
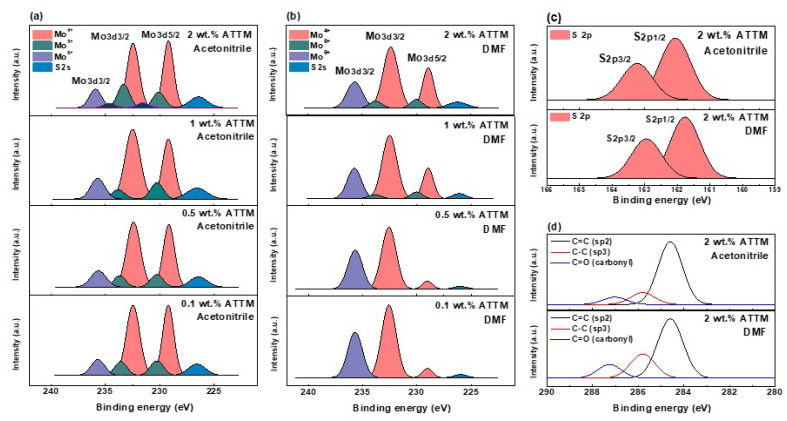
X-ray photoelectron spectra of the MoS_2_–CNW samples: Mo 3d region of (**a**) AM–CNW and (**b**) DM–CNW, (**c**) S 2p region of 2 wt.% ATTM in AM–CNW and DM–CNW, and (**d**) C 1s region of 2 wt.% ATTM in AM–CNW and DM–CNW.

To observe the more significant chemical bonds in the hybrid MoS_2_–CNW active materials, XPS analysis was next performed for the different concentrations of ATTM in organic solvent. Figure 3a,b shows the XPS spectra for Mo 3d region of the MoS_2_ samples synthesized using acetonitrile and DMF solutions with various ATTM weight percentages. The differences in the results for the two different solvents are clearly shown in the different Mo 3d spectra. For the AM–CNW samples, two strong main peaks at 229.2 and 232.5 eV were observed, which can be respectively attributed to the Mo 3d_5/2_ and 3d_3/2_ doublets of the Mo^4+^ oxidation state in the typical binding state of MoS_2_ [53,54]. These two distinctive peaks indicate the presence of 2H–MoS_2_, which is in accordance with the Raman analysis results [55,56]. In addition, the weak peak at 226.5 eV was ascribed to the S 2s of MoS_2_. Another oxidation state of the Mo 3d orbital at 235.7 eV is derived from the 3d_3/2_ binding energy of Mo^6+^, indicating the oxidation level of Mo resulting from MoO_3_ formation. This result was attributed to the thermal-induced etching and oxidation effects of the hydrogen radicals. From the deconvoluted spectra, small amounts of Mo 3d oxidation states (corresponding to Mo^5+^) at 230.3 and 233.6 eV were also observed. These can either be a consequence of the oxidization of the Mo^4+^ state in MoS_2_ into Mo^5+^ and Mo^6+^ oxidation states, owing to the effect of the hydrogen radicals, or transient products from the intermediate ATTM–MoS_2_ transformation process [47,57,58].

For the dimethylformamide samples, the major signals corresponding to the Mo4+ oxidation state were also observed at binding energies of 228.8 and 232.7 eV. However, the peak at 235.7 eV associated with Mo–O binding is predominant below 0.5 wt.% ATTM concentration. As shown in Figure 3b, with the increase in the ATTM concentration in DMF, the Mo 3d_3/2_ of the Mo–O bond decreased and the Mo 3d_5/2_ of the Mo–S bond increased, while the peak at 232.7 eV was retained. Furthermore, the highest peak centered at 232.7 eV can be described as the convolution of two peaks related to the Mo 3d_3/2_ of the Mo–S and Mo 3d_5/2_ of the Mo–O bonds. This result suggests that the Mo–O bond preferentially occurs at the CNW–MoS_2_ interface and then transforms into a Mo–S bond [58,59].

From the SEM images in Figure 1c,d, it can be observed that MoS_2_ is combined in the form of net-like networks between each nanowall of the CNW scaffold. The analysis of the S 2p region of the XPS spectra shows a typical S 2p_3/2_ (162 eV) and S 2p_1/2_ (163 eV) doublet originating from the Mo–S bond in the MoS_2_ phase for both the AM–CNW and DM–CNW samples, as shown in Figure 3c. The C 1 s spectra of the CNW scaffolds were also analyzed using XPS, which revealed dominant distributions of sp2 hybridized carbon atoms at approximately 284.6 eV [60]. The peak at 285.8 eV is associated with sp^3^ C–C binding, which is derived from the carbon buffer layer between the transition metal substrate and CNWs. The buffer layer consisting of amorphous carbon containing sp3 carbon bonds can be developed due to the catalytic effect of the transition metal substrate. Here, the transition metal surface acts as a catalytic decomposition site for the carbon source. Thus, carbon-containing precursors can be easily decomposed on the metal surface to form an amorphous carbon layer in plasma-enhanced chemical vapor deposition (PECVD) systems. The catalytic effect of the transition metal surface promotes rapid absorption of the carbon sources; thus, the time and temperature are insufficient for the crystallization of carbon to occur [61,62,63]. In addition, the small peaks at 287 eV are attributed to C = O (carboxyl) functional groups, based on the presence of open top edges [61,64,65].

From the results of the C 1s spectra and Raman shifts, it is clear that the CNW scaffold remains even after MoS_2_ synthesis by hydrogen radical treatment.

### 3.4. CV Analysis

The interactions at the heterostructural hybrid interface of the MoS_2_–CNWs—based on their surface morphologies and the oxidation states of MoS_2_ over the CNW scaffolds—were expected to differ from those of the pristine CNW electrode. Furthermore, the electrical coupling of the electronically conductive CNW with the redox-active MoS_2_ was anticipated to enhance the capacitive performance of the hybrid electrodes by promoting intercalation and deintercalation of the K^+^ cations in KOH electrolyte during the electrochemical charge transfer mechanism [66]. Thus, the electrochemical capacitive behavior of the MoS_2_–CNW hybrid electrodes was investigated using CV. The CV measurements were performed on a three-electrode system in an aqueous 6 M KOH electrolyte, with Ag/AgCl and Pt as the reference and counter electrodes, respectively. The working potential range of a supercapacitor normally depends on the nature of the active electrode materials and the type of electrolyte. For a KOH solution, the supercapacitor cells can operate within the range of −1–0.5 V [67,68].

From the CV curves, the following analytical formulae were employed to obtain the specific capacitance of the MoS_2_–CNW electrodes:

(1) $$C_{s}=\frac{\int Idv}{V_{s}\times △V\times A}$$

where *∫Idv* is the area under the CV curve, vs. is the scan rate, Δ*V* is the potential window, and *A* is the selected area of electrode.

The energy density was calculated from the equation

(2) $$E_{d}=\frac{1}{2}\times C_{s}\times △V^{2}\times \frac{1000}{3600}$$

where *Cs* is the specific capacitance obtained from Equation (1) and Δ*V* is the applied potential.

The power density was calculated from the equation

(3) $$P_{d}=\frac{1}{2}\times C_{s}\times △V\times V_{s}$$

where *Cs* is the specific capacitance obtained from Equation (1), Δ*V* is the potential window, and vs. is the scan rate.

**Figure 4:**
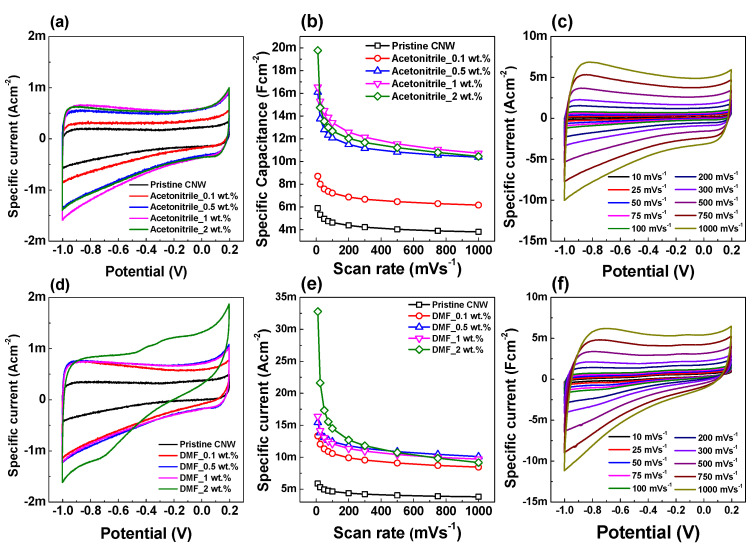
Cyclic voltammograms of the MoS_2_–CNW hybrid electrodes: (**a**) AM–CNW samples at 10 mVs^−1^ scan rate, (**b**) specific capacitance variation with scan rates in the range of 10–1000 mVs^−1^, (**c**) cyclic vol-tammetry (CV) curve of 2 wt.% ATTM in the AM–CNW samples, (**d**) DM–CNW samples at 10 mVs^−1^ scan rate, (**e**) specific capacitance variation with scan rates in the range of 10–1000 mVs^−1^, and (**f**) CV curves of the 2 wt.% ATTM in DM–CNW samples.

The cyclic voltammograms of the hybrid electrodes with different ATTM concentrations and organic solutions are presented in Figure 4. The specific capacitance was enhanced for all of the hybrid supercapacitor cells as compared to that of the pristine CNW (Figure 4a,d).

The cyclic voltammograms of the AM–CNW samples present an archetypal horizontal straight curve without any significant redox peaks within the operating potential range, which implies that the MoS_2_–CNW composites can function as an EDLC and store charge by adsorbing ions on the electrode−electrolyte interface (Figure 4a) [69]. Compared with the capacitance value of 6 mFcm^−2^ for pristine CNW, the specific capacitance of AM–CNW also increased to 8.7, 16, 17, and 20 mFcm^−2^ with increasing ATTM concentration from 0.1 to 2 wt.% at a scan rate 10 mVs^−1^ (Figure 4b). This was attributed to the increase in the effective contact area, as shown in the SEM images of the AM–CNW samples. Figure 4b shows plots of the calculated specific capacitance values with increasing scan rate and ATTM concentration. As the scan rate increased, the specific capacitance value decreased from 8.7, 16, 17, and 20 mFcm^−2^ to 6.2, 10, 11, and 10 mFcm^−2^ for the 0.1, 0.5, 1, and 2 wt.% ATTM concentrations, respectively. Further, as the scan rate was increased from 10 to 1000 mVs^−1^, the CV curves retained a quasi-rectangular shape without any significant deformation. This indicates that AM–CNW works mainly through an electrical double-layer capacitive charge storage mechanism and displays a good fast charge–discharge performance

On the other hand, the 2 wt.% DM–CNW sample (Figure 4d) presented small reversible redox peaks at 0.2 and −0.72 V, indicating that the faradaic process of MoS_2_ contributes to increased capacitance. The two reversible redox peaks were attributed to the oxidation–reduction process of the Mo atoms by the process of Mo^4+^↔Mo^5+^↔Mo^6+^ [70].

The voltammograms of all of the hybrid samples demonstrate excellent stability at high scan rates. Figure 4e shows that the specific capacitance values of the 0.1–2 wt.% DM–CMW samples increased by 13, 15, 16, and 33 mFcm^−2^, respectively, compared to that of pristine CNW, while they decreased with increasing scan rate—from 10–1000 mVs^−1^—to 8.5, 10, 9.7, and 9.2 mFcm^−2^, respectively. As the scan rate increased to 1000 mVs^−1^, the small redox peaks were barely visible, as shown in Figure 4f. For the redox species, the peak current *i_p_* (A) is described by Randles–Sevcik equation;

(4) $$i_{p}=0.446nFAC^{0}{\left(\frac{nFvD_{o}}{RT}\right)}^{1/2}$$

where, *v* is the scan rate (Vs^−1^), *n* is the number of electrons transferred in redox event, *A* is the electrode surface area (cm^2^), *D_o_* is the diffusion coefficient of the analyte, *C^o^* is the bulk concentration of the analyte, *F* is the Faraday’s constant, *R* is the universal gas constant and *T* is the temperature. Faster scan rate decreases the diffusion layer, which controls mass transfer of K^+^ cations. Thus, the peak current increases linearly with the square root of scan rate (*v*), as expressed in Equation (4). In contrast, for the electrode-adsorbed species, the peak current can be described as the following equation;

(5) $$i_{p}=\frac{n^{2}F^{2}}{4RT}vA\Gamma^{*}$$

where, Γ* is the surface coverage when the adsorbed species is represented in mol cm^−2^. As described in Equation (5), the peak current increases linearly with scan rate (*v*) [71].

Since the data were collected from various literatures, which employed different parameters (e.g., scan rate, mass of active material, type of electrolyte, two/three-electrode systems) and devices (e.g., active electrode half-cell or two-electrode full-cell), it is difficult to directly compare all parameters of the electrode materials. Therefore, the detailed information regarding electrode active materials, electrolytes, and capacitance values from the original reports are listed in Table 1 as a reference for comparison.

### 3.5. Ragone Plots

The energy and power densities of electrochemical capacitor electrodes are also important considerations for estimating the capacitor performance. Hence, the energy and power densities were next calculated from the CV results of the MoS_2_–CNW samples, to evaluate the heterostructure MoS_2_–CNW electrode performance.

**Figure 5:**
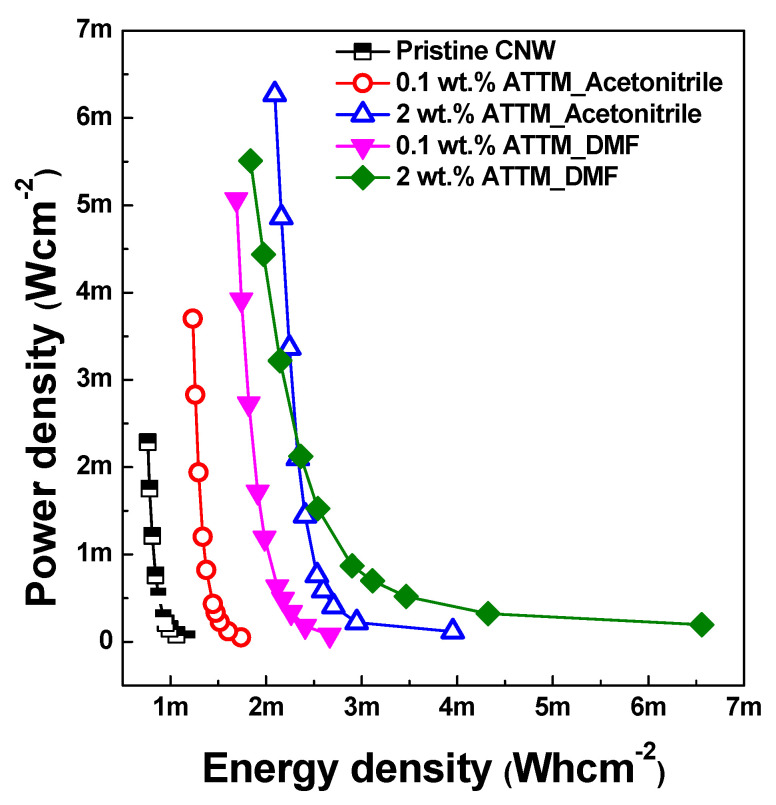
Ragone plot of the MoS_2_–CNW hybrid electrode.

Figure 5 shows the Ragone plots of the AM–CNW and DM–CNW samples compared to that of the pristine CNW electrode. As the pristine CNW power density increased from 35.3 μWcm^−2^ to 2.29 mWcm^−2^, the energy density decreased from 1.18 mWhcm^−2^ to 0.76 mWhcm^−2^ when the scan rate was increased from 10 mVs^−1^ to 1000 mVs^−1^. Compared to that of pristine CNW, all of the MoS_2_–CNW hybrid electrodes exhibited enhanced capacitive performances. Thus, for the 0.1AM–CNW sample, the power and energy densities varied from 52.2 μWcm^−2^ to 3.7 mWcm^−2^ and from 1.75 m to 1.23 mWhcm^−2^, respectively, and for 2AM–CNW, from 120 μWcm^−2^ to 6.27 mWcm^−2^ and from 3.95 mWhcm^−2^ to 2.69 mWhcm^−2^, respectively. In the case of the DM–CNW samples, the power and energy densities for the 0.1DM–CNW sample varied from 80 μWcm^−2^ to 5.1 mWcm^−2^ and from 2.67 mWhcm^−2^ to 1.69 mWhcm^−2^, respectively, and for the 2DM–CNW sample, from 19.7 μWcm^−2^ to 5.5 mWcm^−2^ and from 6.56 mWhcm^−2^ to 1.84 mWhcm^−2^, respectively.

The Ragone plots clearly show that the decrease in energy density slightly increased with the increase in the specific capacitance and scan rate. This was attributed to the nature of the correlations between the ions in the electrolytes and electrode materials in the supercapacitor. Particularly, while the 2DM–CNW sample possesses a much higher energy density than that of 2AM–CNW at a low scan rate, its capacitive performance deterioration was greater than that of 2AM–CNW at a high scan rate. Owing to the restricted temporal limits, a lower scan rate for the electrode provides a larger specific capacitance due to the effective contact between the electrolyte ions and electrode, which is considerably reduced with an increase in the scan rate [84]. In the case of 2DM–CNW, the decrease was greatly increased owing to the pseudocapacitance caused by the redox reaction of Mo.

### 3.6. EIS Analysis

**Figure 6:**
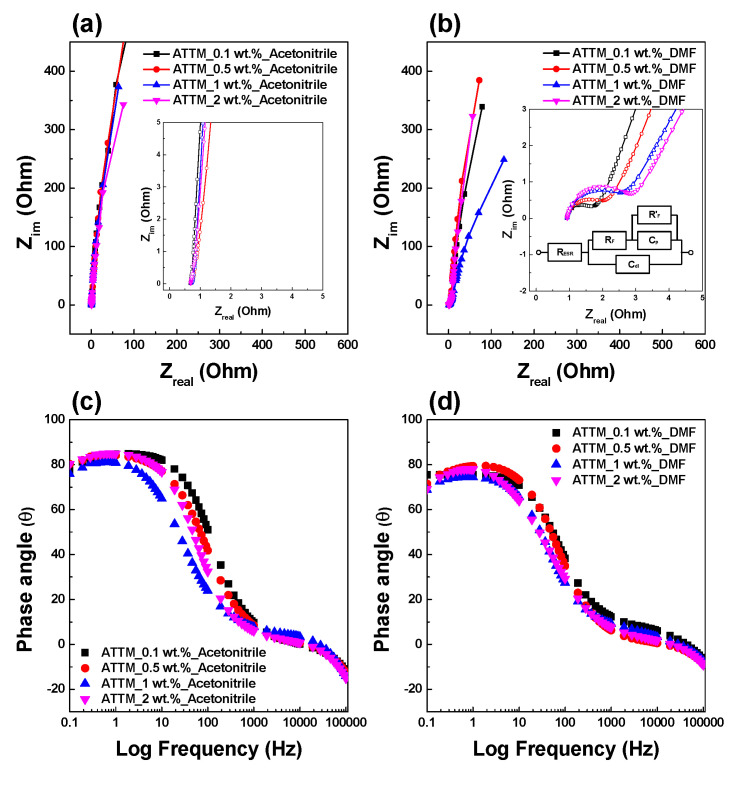
Nyquist plots of the (**a**) AM–CNW and (**b**) DM–CNW samples and Bode plots of the (**c**) AM–CNW and (**d**) DM–CNW samples.

To investigate the frequency response properties at the interface between the electrolyte and heterostructure electrode surface of the hybrid MoS_2_–CNW capacitors, EIS was performed in the frequency range of 100 mHz–100 kHz. Figure 6a,b shows the Nyquist plots of the AM–CNW and DM–CNW samples with varied ATTM concentrations, analyzed using a DC bias potential of 0.5 V at open circuit potential. As revealed in Figure 6a, only a near-vertical line was observed for the entire frequency region, without the semicircle in the high-frequency region. This indicates that the AM–CNW capacitors utilize EDLC charge storage mechanism with low ion diffusion resistance [13,85,86].

The absence of semicircles at the intersections of the Nyquist plots indicates ohmic contact between the CNWs and nickel current collectors. The high carbon solubility of nickel substrates (compared to those of the other transition metals) promotes ohmic contact with the CNW [85,86]. In contrast, for the DM–CNW samples, the semicircles gradually increased with ATTM concentration at high frequencies, indicating high ionic resistance [87]. This is due to the impoverished electrical and ionic conductivities between two adjacent S–Mo–S units [88,89]. These results are in good agreement with the XPS results shown in Figure 3b. The equivalent circuit, considering double-layer capacitance (*C_dl_*) and pseudocapacitance (*C_p_*) separately, is provided in Figure 6b. In the circuit, *R_ESR_* corresponds to the aggregated equivalent serial resistance of the electrical resistances of the electrodes and the ionic resistance of the electrolyte; *R_F_* corresponds to the Faradaic charge transfer resistance and *R’_F_* represents the leakage through the Faradaic reactions.

The pseudocapacitive characteristics of the DM–CNW samples were additionally confirmed using Bode phase angle plots (Figure 6c,d). For ideal capacitors, Bode plots represent a 90° phase angle shift in the low-frequency region [86]. For ATTM concentrations of 0.1, 0.5, 1, and 2 wt.%, the AM–CNW samples presented respective phase angles of 78°, 80°, 76°, and 80°, which approached ideal EDLC capacitive behavior. In contrast, the phase angles of the DM–CNW samples declined to 75°, 71°, 68°, and 69°, respectively, confirming the existence of psuedocapacitance properties.

### 3.7. Life-Cycle Testing

**Figure 7:**
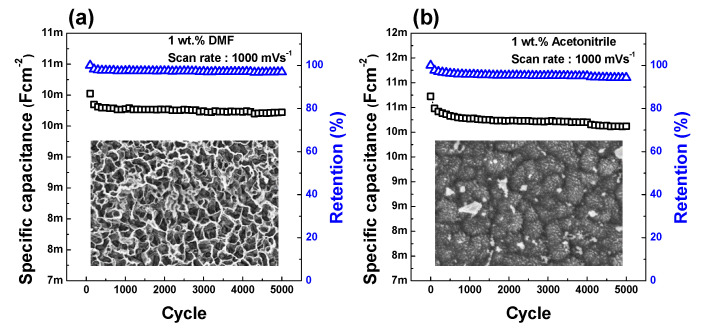
Life cycle tests of the MoS_2_–CNW samples in 1 wt.% (**a**) DMF and (**b**) acetonitrile for up to 5000 cycles and surface morphology change after life cycle test.

Figure 7a,b shows the results of life cycle test for the AM–CNW and DM–CNW samples, respectively. Cycle stability tests were performed using 1 wt.% ATTM concentration samples at a 1000 mVs^−1^ scan rate, for 5000 cycles, using CV measurement.

As shown in Figure 7a,b, the specific capacitance drop during the initial 100 cycles is caused by the small volume change of MoS_2_ during the charge and discharge processes [90,91]. After cycling, the specific capacitances of the AM–CNW and DM–CNW samples decreased from 10.7 mFcm^−2^ to 10.1 mFcm^−2^ and 10 mFcm^−2^ to 9.7 mFcm^−2^, respectively, retaining 94% and 97% of the initial specific capacitances, respectively. These results indicate the outstanding cycle stability of the as-produced hybrid capacitors. Also, the surface morphologies are barely deteriorated after life cycle test. The results indicate the hybrid electrodes possess excellent structural stability.

## 4. Conclusions

The synthesis of CNW-based MoS_2_ hybrid electrodes for EDLC was investigated through experimental analyses using various concentrations of ATTM in DMF and acetonitrile solutions, under remote-plasma-providing hydrogen radicals. The ATTM–MoS_2_ transformation and crystallization by introducing hydrogen radicals were successfully accomplished without deterioration of the CNW scaffold in a short time.

The results revealed that ATTM–MoS_2_ crystallization can be achieved by hydrogen radicals in the absence of a heating system. Furthermore, the electrochemical characteristics depending on the MoS_2_ concentration were investigated and compared with those of the pristine CNW electrode. Electrochemical investigations revealed that MoS_2_ acts as a Faradaic material. Thus, the highest capacitance of 33 mFcm^−2^ was achieved using 2 wt.% MoS_2_ with DMF. Further enhancement of the specific capacitance in DMF was influenced by both the MoS_2_ concentration and morphology of the synthesized MoS_2_ on the CNW scaffold. EIS analysis revealed that the MoS_2_-coated CNW electrodes can operate as stable EDLC electrodes at a rapid charging–discharging rate of 1000 mVs^−1^. The Nyquist and Bode plots revealed that the MoS_2_-coated CNW electrodes behave as EDLCs with partially included pseudocapacitance. The life cycle test was successfully performed for 5000 cycles without a significant capacitance drop, and retained capacitances of 94% and 97% were achieved for acetonitrile and DMF, respectively. The method of synthesizing MoS_2_ through hydrogen radical publication presented in this paper should contribute to the mass production process since MoS_2_ can be synthesized in a very short time.

## Figures and Tables

**Table 1:** Detailed information regarding EDLC electrodes.

Materials	Syn. Route	Electrolyte	Areal Capacitance (mFcm^−2^)	Ref.
Carbon allotropes				
N-doped CNW	ECR plasma	1 M Na_2_SO_4_	5.8 (at 100 mV s^−^^1^)	[15]
Defected few layer graphene	CVD/Ar+ plasma etching	0.25 M TEABF_4_	0.05 (at 1000 mV s^−^^1^)	[72]
Reduced GO/CNTs	Electrostatic spray deposition	3 M KCl	6.1 (at 100 mV s^−^^1^)	[73]
Reduced graphene oxide	CVD	PVA-H_3_PO_4_	0.394 (at 1–100 mV s^−^^1^)	[74]
Activated graphene N-doped	Microwave exfoliation	6 M KOH	0.006 (at 20 mV s^−^^1^)	[75]
MoS_2_-homogeneous				
MoS_2_ nanosheets	Hydrothermal synthesis	NaOH	8 (at 10 mV s^−^^1^)	[76]
Porous MoS_2_	RF sputtering	0.5 M H_2_SO_4_	33 (at 25.47 mA cm^−2^)	[77]
Edge-oriented MoS_2_	CVD	0.5 M H_2_SO_4_	12.5 (at 50 mV s^−^^1^)	[45]
MoS_2_ nanoflower	Hydrothermal synthesis	0.5 M H_2_SO_4_	290 (at 1 mA cm^−^^2^)	[78]
MoS_2_-Carbon composites				
MoS_2_-graphene	Liquid phase exfoliation	1 M Na_2_SO_4_	4.29 (at 5 mV s^−^^1^)	[79]
Ag nanowire-MoS_2_	Solution based synthesis	PVA-H_2_SO_4_	27 (at 200 mV s^−^^1^)	[80]
Carbon-MoS_2_-Carbon Nanoplates	Hydrothermal synthesis	1 M LiSO_4_	0.12 (at 0.1 A g^−1^)	[81]
MoS_2_/rGO	Hydrothermal synthesis	1 M H_2_SO_4_	14.09 (at 5 mV s^−^^1^)	[82]
MoS_2_@CNT/RGO	Hydrothermal and vacuum filtration	1 M H_2_SO_4_	129 (at 0.1 mA cm^−2^)	[83]
MoS_2_/CNW composites	Remote plasma	6 M KOH	33 (at 10 mV s^−^^1^)	This work

## Data Availability

The data presented in this study are available on request from the first or corresponding author.

## References

[1] Xiong G., Meng C., Reifenberger R.G., Irazoqui P.P., Fisher T.S. (2014). A Review of Graphene-Based Electrochemical Microsupercapacitors. Electroanalysis.

[2] Kötz R., Carlen M. (2000). Principles and Applications of Electrochemical Capacitors. Electrochim. Acta.

[3] Costentin C., Porter T.R., Savéant J.M. (2017). How Do Pseudocapacitors Store Energy? Theoretical Analysis and Experimental Illustration. ACS Appl. Mater. Interfaces.

[4] Soon J.M., Loh K.P. (2007). Electrochemical Double-Layer Capacitance of MoS_2_ Nanowall Films. Electrochem. Solid State Lett..

[5] Zhang Y., Feng H., Wu X., Wang L., Zhang A., Xia T., Dong H., Li X., Zhang L. (2009). Progress of Electrochemical Capacitor Electrode Materials: A Review. Int. J. Hydr. Energy.

[6] Chuang C.-M., Huang C.-W., Teng H., Ting J.-M. (2010). Effects of Carbon Nanotube Grafting on the Performance of Electric Double Layer Capacitors. Energy Fuels.

[7] Zhu Y., Murali S., Stoller M.D., Ganesh K.J., Cai W., Ferreira P.J., Pirkle A., Wallace R.M., Cychosz K.A., Thommes M. (2011). Carbon-Based Supercapacitors Produced by Activation of Graphene. Science.

[8] Zhang L.L., Zhao X.S. (2009). Carbon-Based Materials as Supercapacitor Electrodes. Chem. Soc. Rev..

[9] Pandolfo A.G., Hollenkamp A.F. (2006). Carbon Properties and Their Role in Supercapacitors. J. Power Sources.

[10] Pech D., Brunet M., Durou H., Huang P., Mochalin V., Gogotsi Y., Taberna P.L., Simon P. (2010). Ultrahigh-Power Micrometre-Sized Supercapacitors Based on Onion-Like Carbon. Nat. Nanotechnol..

[11] Wu Z.S., Parvez K., Feng X., Müllen K. (2013). Graphene-Based in-Plane Micro-Supercapacitors with High Power and Energy Densities. Nat. Commun..

[12] Pech D., Brunet M., Taberna P.-L., Simon P., Fabre N., Mesnilgrente F., Conédéra V., Durou H. (2010). Elaboration of a Microstructured Inkjet-Printed Carbon Electrochemical Capacitor. J. Power Sources.

[13] Liu C.-C., Tsai D.-S., Chung W.-H., Li K.-W., Lee K.-Y., Huang Y.-S. (2011). Electrochemical Micro-Capacitors of Patterned Electrodes Loaded with Manganese Oxide and Carbon Nanotubes. J. Power Sources.

[14] Cheng Q., Tang J., Ma J., Zhang H., Shinya N., Qin L.C. (2011). Graphene and Carbon Nanotube Composite Electrodes for Supercapacitors with Ultra-High Energy Density. Phys. Chem. Chem. Phys..

[15] Shin J.H., Park H.J., Song Y.i., Choi Y.S., Suh S.-J. (2020). Morphological Optimization and Nitrogen Functionalization of Vertically Oriented CNW for High Performance Electrical Double Layer Capacitor Electrode. Electrochim. Acta.

[16] Cai M., Outlaw R.A., Quinlan R.A., Premathilake D., Butler S.M., Miller J.R. (2014). Fast Response, Vertically Oriented Graphene Nanosheet Electric Double Layer Capacitors Synthesized from C_2_H_2_. ACS Nano.

[17] Gao W., Singh N., Song L., Liu Z., Reddy A.L., Ci L., Vajtai R., Zhang Q., Wei B., Ajayan P.M. (2011). Direct Laser Writing of Micro-Supercapacitors on Hydrated Graphite Oxide Films. Nat. Nanotechnol..

[18] Yang W., Ni M., Ren X., Tian Y., Li N., Su Y., Zhang X. (2015). Graphene in Supercapacitor Applications. Curr. Opin. Colloid Interface Sci..

[19] Lin L., Lei W., Zhang S., Liu Y., Wallace G.G., Chen J. (2019). Two-Dimensional Transition Metal Dichalcogenides in Supercapacitors and Secondary Batteries. Energy Storage Mater..

[20] Choi W., Choudhary N., Han G.H., Park J., Akinwande D., Lee Y.H. (2017). Recent Development of Two-Dimensional Transition Metal Dichalcogenides and Their Applications. Mater. Today.

[21] Chhowalla M., Shin H.S., Eda G., Li L.J., Loh K.P., Zhang H. (2013). The Chemistry of Two-Dimensional Layered Transition Metal Dichalcogenide Nanosheets. Nat. Chem..

[22] Kato K., Sayed F.N., Babu G., Ajayan P.M. (2018). All 2D Materials as Electrodes for High Power Hybrid Energy Storage Applications. 2D Mater..

[23] Choudhary N., Islam M.A., Kim J.H., Ko T.-J., Schropp A., Hurtado L., Weitzman D., Zhai L., Jung Y. (2018). Two-Dimensional Transition Metal Dichalcogenide Hybrid Materials for Energy Applications. Nano Today.

[24] Li H., Yang Q., Mo F., Liang G., Liu Z., Tang Z., Ma L., Liu J., Shi Z., Zhi C. (2019). MoS_2_ Nanosheets with Expanded Interlayer Spacing for Rechargeable Aqueous Zn-Ion Batteries. Energy Storage Mater..

[25] Yu D., Pang Q., Gao Y., Wei Y., Wang C., Chen G., Du F. (2018). Hierarchical Flower-Like VS_2_ Nanosheets—A High Rate-Capacity and Stable Anode Material for Sodium-Ion Battery. Energy Storage Mater..

[26] Voiry D., Salehi M., Silva R., Fujita T., Chen M., Asefa T., Shenoy V.B., Eda  G., Chhowalla M. (2013). Conducting MoS_2_ nanosheets as catalysts for hydrogen evolution reaction. Nano Lett..

[27] Li Z., Meng X., Zhang Z. (2018). Recent development on MoS_2_-based photocatalysis: A review. J. Photochem. Photobiol. C Photochem. Rev..

[28] Pham T., Li G., Bekyarova E., Itkis M.E., Mulchandani A. (2019). MoS_2_-based optoelectronic gas sensor with sub-parts-per-billion limit of NO_2_ gas detection. ACS Nano.

[29] Kumar R., Zheng W., Liu X., Zhang J., Kumar M. (2020). MoS_2_-based nanomaterials for room-temperature gas sensors. Adv. Mater. Technol..

[30] Minakshi M., Mitchell D.R., Munnangi A.R., Barlow A.J., Fichtner M. (2018). New insights into the electrochemistry of magnesium molybdate hierarchical architectures for high performance sodium devices. Nanoscale.

[31] Sundaram M.M., Appadoo D. (2020). Traditional salt-in-water electrolyte vs. water-in-salt electrolyte with binary metal oxide for symmetric supercapacitors: Capacitive vs. faradaic. Dalton Trans..

[32] Zhao F., Lin J., Lei Z., Yi Z., Qin F., Zhang J., Liu L., Wu X., Yang W., Wu P. (2020). Realization of 18.97% theoretical efficiency of 0.9 μm thick c-Si/ZnO heterojunction ultrathin-film solar cells via surface plasmon resonance enhancement. Phys. Chem. Chem. Phys..

[33] Zhong M.E., Guan J., Feng Q., Wu X., Xiao Z., Zhang W., Tong S., Zhou N., Gong D. (2018). Accelerated polysulfide redox kinetics revealed by ternary sandwich-type S@ Co/N-doped carbon nanosheet for high-performance lithium-sulfur batteries. Carbon.

[34] Kong P., Zhu L., Li F., Xu G. (2019). Self-Supporting Electrode Composed of SnSe Nanosheets, Thermally Treated Protein, and Reduced Graphene Oxide with Enhanced Pseudocapacitance for Advanced Sodium-Ion Batteries. ChemElectroChem.

[35] Shen Y., Wang X., Hu H., Jiang M., Bai Y., Yang X., Shu H. (2015). Sheet-like structure FeF 3/graphene composite as novel cathode material for Na ion batteries. RSC Adv..

[36] Krishnan U., Kaur M., Singh K., Kumar M., Kumar A. (2019). A Synoptic Review of MoS_2_: Synthesis to Applications. Superlattices Microstruct..

[37] Gupta D., Chauhan V., Kumar R. (2020). A Comprehensive Review on Synthesis and Applications of Molybdenum Disulfide (MoS_2_) Material: Past and Recent Developments. Inorg. Chem. Commun..

[38] Brito J.L., Ilija M., Hernández P.H. (1995). Thermal and Reductive Decomposition of Ammonium Thiomolybdates. Thermochim. Acta.

[39] Shuai X., Bo Z., Kong J., Yan J., Cen K. (2017). Wettability of Vertically Oriented Graphenes with Different Intersheet Distances. RSC Adv..

[40] Zang J., Ryu S., Pugno N., Wang Q., Tu Q., Buehler M.J., Zhao X. (2013). Multifunctionality and Control of the Crumpling and Unfolding of Large-Area Graphene. Nat. Mater..

[41] Cassie A.B.D., Baxter S. (1944). Wettability of Porous Surfaces. Trans. Faraday Soc..

[42] Tuinstra F., Koenig J.L. (1970). Raman Spectrum of Graphite. J. Chem. Phys..

[43] Bokobza L., Bruneel J.-L., Couzi M. (2015). Raman Spectra of Carbon-Based Materials (from Graphite to Carbon Black) and of Some Silicone Composites. C.

[44] Ni Z.H., Fan H.M., Feng Y.P., Shen Z.X., Yang B.J., Wu Y.H. (2006). Raman Spectroscopic Investigation of Carbon Nanowalls. J. Chem. Phys..

[45] Yang Y., Fei H., Ruan G., Xiang C., Tour J.M. (2014). Edge-Oriented MoS_2_ Nanoporous Films as Flexible Electrodes for Hydrogen Evolution Reactions and Supercapacitor Devices. Adv. Mater..

[46] Verble J.L., Wieting T.J. (1970). Lattice Mode Degeneracy in MoS_2_ and Other Layer Compounds. Phys. Rev. Lett..

[47] Liang L., Meunier V. (2014). First-Principles Raman Spectra of MoS_2_, WS_2_ and Their Heterostructures. Nanoscale.

[48] Jung C., Yang H.I., Choi W. (2019). Effect of Ultraviolet-Ozone Treatment on MoS_2_ Monolayers: Comparison of Chemical-Vapor-Deposited Polycrystalline Thin Films and Mechanically Exfoliated Single Crystal Flakes. Nanoscale Res. Lett..

[49] Zhu J., Wang Z., Yu H., Li N., Zhang J., Meng J., Liao M., Zhao J., Lu X., Du L. (2017). Argon Plasma Induced Phase Transition in Monolayer MoS_2_. J. Am. Chem. Soc..

[50] Ding Q., Meng F., English C.R., Cabán-Acevedo M., Shearer M.J., Liang D., Daniel A.S., Hamers R.J., Jin S. (2014). Efficient Photoelectrochemical Hydrogen Generation Using Heterostructures of Si and Chemically Exfoliated Metallic MoS_2_. J. Am. Chem. Soc..

[51] Jiménez Sandoval S.S., Yang D., Frindt R.F., Irwin J.C. (1991). Raman Study and Lattice Dynamics of Single Molecular Layers of MoS_2_. Phys. Rev. B Condens. Matter..

[52] Gao J., Li L., Tan J., Sun H., Li B., Idrobo J.C., Singh C.V., Lu T.M., Koratkar N. (2016). Vertically Oriented Arrays of ReS_2_ Nanosheets for Electrochemical Energy Storage and Electrocatalysis. Nano Lett..

[53] Kim K.S., Kim K.H., Nam Y., Jeon J., Yim S., Singh E., Lee J.Y., Lee S.J., Jung Y.S., Yeom G.Y. (2017). Atomic Layer Etching Mechanism of MoS_2_ for Nanodevices. ACS Appl. Mater. Interfaces.

[54] Altavilla C., Sarno M., Ciambelli P. (2011). A Novel Wet Chemistry Approach for the Synthesis of Hybrid 2D Free-Floating Single or Multilayer Nanosheets of MS_2_@oleylamine (M═Mo, W). Chem. Mater..

[55] Yao Y., Ao K., Lv P., Wei Q. (2019). MoS_2_ Coexisting in 1T and 2H Phases Synthesized by Common Hydrothermal Method for Hydrogen Evolution Reaction. Nanomaterials.

[56] Eda G., Yamaguchi H., Voiry D., Fujita T., Chen M., Chhowalla M. (2011). Photoluminescence from Chemically Exfoliated MoS_2_. Nano Lett..

[57] Li X., Li X., Zang X., Zhu M., He Y., Wang K., Xie D., Zhu H. (2015). Role of Hydrogen in the Chemical Vapor Deposition Growth of MoS_2_ Atomic Layers. Nanoscale.

[58] Wang H.W., Skeldon P., Thompson G.E. (1997). XPS Studies of MoS_2_ Formation from Ammonium Tetrathiomolybdate Solutions (3). Surf. Coat. Technol..

[59] da Silveira Firmiano E.G., Rabelo A.C., Dalmaschio C.J., Pinheiro A.N., Pereira E.C., Schreiner W.H., Leite E.R. (2014). Supercapacitor Electrodes Obtained by Directly Bonding 2D MoS_2_ on Reduced Graphene Oxide. Adv. Energy Mater..

[60] Evlashin S.A., Maksimov Y.M., Dyakonov P.V., Pilevsky A.A., Maslakov K.I., Mankelevich Y.A., Voronina E.N., Vavilov S.V., Pavlov A.A., Zenova E.V. (2019). N-Doped Carbon Nanowalls for Power Sources. Sci. Rep..

[61] Rybin M., Pereyaslavtsev A., Vasilieva T., Myasnikov V., Sokolov I., Pavlova A., Obraztsova E., Khomich A., Ralchenko V., Obraztsova E. (2016). Efficient Nitrogen Doping of Graphene by Plasma Treatment. Carbon.

[62] Zhao J., Shaygan M., Eckert J., Meyyappan M., Rümmeli M.H. (2014). A Growth Mechanism for Free-Standing Vertical Graphene. Nano Lett..

[63] Zhu M., Wang J., Holloway B.C., Outlaw R.A., Zhao X., Hou K., Shutthanandan V., Manos D.M. (2007). A Mechanism for Carbon Nanosheet Formation. Carbon.

[64] Ederer J., Janoš P., Ecorchard P., Tolasz J., Štengl V., Beneš H., Perchacz M., Pop-Georgievski O. (2017). Determination of Amino Groups on Functionalized Graphene Oxide for Polyurethane Nanomaterials: XPS Quantitation vs. Functional Speciation. RSC Adv..

[65] Lu Y.F., Lo S.T., Lin J.C., Zhang W., Lu J.Y., Liu F.H., Tseng C.M., Lee Y.H., Liang C.T., Li L.J. (2013). Nitrogen-Doped Graphene Sheets Grown by Chemical Vapor Deposition: Synthesis and Influence of Nitrogen Impurities on Carrier Transport. ACS Nano.

[66] Zhou R., Wei S., Liu Y., Gao N., Wang G., Lian J., Jiang Q. (2019). Charge Storage by Electrochemical Reaction of Water Bilayers Absorbed on MoS_2_ Monolayers. Sci. Rep..

[67] Hekmat F., Sohrabi B., Rahmanifar M.S., Vaezi M.R. (2014). Supercapacitive Properties of Coiled Carbon Nanotubes Directly Grown on Nickel Nanowires. J. Mater. Chem. A.

[68] Ali G.A.M., Megiel E., Romański J., Algarni H., Chong K.F. (2018). A Wide Potential Window Symmetric Supercapacitor by TEMPO Functionalized MWCNTs. J. Mol. Liq..

[69] Wei S., Zhou R., Wang G. (2019). Enhanced Electrochemical Performance of Self-Assembled Nanoflowers of MoS_2_ Nanosheets as Supercapacitor Electrode Materials. ACS Omega.

[70] Vincent S.P. (1979). Oxidation–Reduction Potentials of Molybdenum and Iron–Sulphur Centres in Nitrate Reductase from Escherichia coli. Biochem. J..

[71] Elgrishi N., Rountree K.J., McCarthy B.D., Rountree E.S., Eisenhart T.T., Dempsey J.L. (2017). A practical beginner’s guide to cyclic voltammetry. J. Chem. Edu..

[72] Zhu J., Childress A.S., Karakaya M., Dandeliya S., Srivastava A., Lin Y., Rao A.M., Podila R. (2016). Defect-engineered graphene for high-energy-and high-power-density supercapacitor devices. Adv. Mater..

[73] Beidaghi M., Wang C. (2012). Micro-supercapacitors based on interdigital electrodes of reduced graphene oxide and carbon nanotube composites with ultrahigh power handling performance. Adv. Funct. Mater..

[74] Yoo J.J., Balakrishnan K., Huang J., Meunier V., Sumpter B.G., Srivastava A., Conway M., Reddy A.L.M., Yu J., Ajayan P.M. (2011). Ultrathin planar graphene supercapacitors. Nano Lett..

[75] Zhang L.L., Zhao X., Ji H., Stoller M.D., Lai L., Murali S., Mcdonnell S., Cleveger B., Wallace R.M., Ruoff R.S. (2012). Nitrogen doping of graphene and its effect on quantum capacitance, and a new insight on the enhanced capacitance of N-doped carbon. Energy Environ. Sci..

[76] Cao L., Yang S., Gao W., Liu Z., Gong Y., Ma L., Shi G., Lei S., Zhang Y., Zhang S. (2013). Direct laser-patterned micro-supercapacitors from paintable MoS_2_ films. Small.

[77] Choudhary N., Patel M., Ho Y.H., Dahotre N.B., Lee W., Hwang J.Y., Choi W. (2015). Directly deposited MoS_2_ thin film electrodes for high performance supercapacitors. J. Mater. Chem. A.

[78] Liu X., Liu L., Wu Y., Wang Y., Yang J., Wang Z. (2019). Rosette-like MoS_2_ nanoflowers as highly active and stable electrodes for hydrogen evolution reactions and supercapacitors. RSC Adv..

[79] Bissett M.A., Kinloch I.A., Dryfe R.A. (2015). Characterization of MoS_2_–graphene composites for high-performance coin cell supercapacitors. ACS Appl. Mater. Interfaces.

[80] Li J., Shi Q., Shao Y., Hou C., Li Y., Zhang Q., Wang H. (2019). Cladding nanostructured AgNWs-MoS_2_ electrode material for high-rate and long-life transparent in-plane micro-supercapacitor. Energy Storage Mater..

[81] Quan T., Goubard-Bretesché N., Härk E., Kochovski Z., Mei S., Pinna N., Ballauff M., Lu Y. (2019). Highly dispersible hexagonal carbon–MoS_2_–carbon nanoplates with hollow sandwich structures for supercapacitors. Chem. Eur. J..

[82] Dutta S., De S. (2018). MoS_2_ nanosheet/rGO hybrid: An electrode material for high performance thin film supercapacitor. Mater. Today Proc..

[83] Wang S., Zhu J., Shao Y., Li W., Wu Y., Zhang L., Hao X. (2017). Three-dimensional MoS_2_@ CNT/RGO network composites for high-performance flexible supercapacitors. Chem. Eur. J..

[84] Pande S.A., Pandit B., Sankapal B.R. (2018). Facile Chemical Route for Multiwalled Carbon Nanotube/Mercury Sulfide Nanocomposite: High Performance Supercapacitive Electrode. J. Colloid Interface Sci..

[85] Ghosh S., Sahoo G., Polaki S.R., Krishna N.G., Kamruddin M., Mathews T. (2017). Enhanced Supercapacitance of Activated Vertical Graphene Nanosheets in Hybrid Electrolyte. J. Appl. Phys..

[86] Bo Z., Wen Z., Kim H., Lu G., Yu K., Chen J. (2012). One-Step Fabrication and Capacitive Behavior of Electrochemical Double Layer Capacitor Electrodes Using Vertically Oriented Graphene Directly Grown on Metal. Carbon.

[87] Xia X., Tu J., Mai Y., Chen R., Wang X., Gu C., Zhao X. (2011). Graphene Sheet/Porous NiO Hybrid Film for Supercapacitor Applications. Chemistry.

[88] Sun Q., Ren Q.-Q., Li H., Fu Z.-W. (2011). High Capacity Sb_2_O_4_ Thin Film Electrodes for Rechargeable Sodium Battery. Electrochem. Commun..

[89] Sun Y., Hu X., Luo W., Huang Y. (2011). Self-Assembled Hierarchical MoO_2_/Graphene Nanoarchitectures and Their Application as a High-Performance Anode Material for Lithium-Ion Batteries. ACS Nano.

[90] Wang J., Wu Z., Hu K., Chen X., Yin H. (2015). High Conductivity Graphene-Like MoS_2_/Polyaniline Nanocomposites and Its Application in Supercapacitor. J. Alloys Compd..

[91] Ali G.A.M., Thalji M.R., Soh W.C., Algarni H., Chong K.F. (2020). One-Step Electrochemical Synthesis of MoS_2_/Graphene Composite for Supercapacitor Application. J. Solid State Electrochem..

